# Supplementary material for: Clinical Risk Factors and Microbiological and Intestinal Characteristics of Carbapenemase-Producing Enterobacteriaceae Colonization and Subsequent Infection
Source: Microbiol Spectr. 2022 Nov 29;10(6):e01906-21. doi: 10.1128/spectrum.01906-21 (PMC9769896; doi:10.1128/spectrum.01906-21)
Supplement: Supplemental file 1 — Supplemental material. Download spectrum.01906-21-s0001.pdf, PDF file, 0.2 MB [file spectrum.01906-21-s0001.pdf]

Supplementary Tab 1. List of primer for detection of genes used in this study.

**Target genes**	**Primer name**	**Sequence 5'-3'**	**Size/Annealing temp**
KPC-2	bla *KPC-2* -F	GCTACACCTAGCTCCACCTTC	989bp/53℃
	bla *KPC-2* -R	ACAGTGGTTGGTAATCCATGC	
NDM-1	bla *NDM-1*-F	CAGCACACTTCCTATCTC	292bp/52℃
	bla *NDM-1*-R	CCGCAACCATCCCCTCTT	
IMP	bla *IMP*-F	CTACCGCAGCAGAGTCTTTG	587bp/58℃
	bla *IMP*-R	AACCAGTTTTGCCTTACCAT	
VIM-2	bla *VIM-2*-F	GTAAATTATTGGTCATTTGACCGTC	743bp/52℃
	bla *VIM-2*-R	CGTTGTGTGCTTGAGCAAGTC	
OXA-48	bla *OXA-48*-F	TTGGTGGCATCGATTATCGG	744bp/58℃
	bla *OXA-48*-R	GAGCACTTCTTTTGTGATGGC	

Supplementary Tab 2. Univariate analysis of risk factors for CRE colonization.

Risk factors	CRE rectal carriers (N=54)	non-colonized patients (N=320)	*P* value (OR[CI 95%])
Age, years			
18-60	22 (40.7)	152 (47.5)	0.357 (0.760 [0.423-1.365])
60-80	15 (27.8)	130 (40.6)	0.073 (0.562 [0.298-1.062])
>80	16 (29.6)	28 (8.8)	<0.001 (4.391 [2.178-8.852])
Sex, male	36 (66.7)	169 (52.8)	0.058 (1.787 [0.974-3.279])
Hospitalization history in the 6 months before screening	37(68.5)	190 (59.4)	0.203 (1.489 [0.804-2.757])
3 or more co-morbid conditions	28 (51.9)	42 (13.1)	<0.001 (7.128[3.817-13.313])
APACHE II score≥21	33 (61.1)	111 (34.7)	<0.001 (2.959[1.634-5.357])
Carriage or infection with another MDRO	32 (59.3)	31 (9.7)	<0.001(13.560[7.028-26.162])
Invasive procedures	31 (57.4)	180 (56.3)	0.874 (1.048[0.585-1.878])
Carbapenem use	15 (27.8)	32 (10.0)	<0.001 (3.462 [1.721-6.961])
Cefoperazone/sulbactam use	14(25.9)	83(25.9)	0.999 (0.999 [0.518-1.930])
Piperacillin/tazobactam use	6(11.1)	51(15.9)	0.361 (0.659[0.268-1.622])
Vancomycin use	6(11.1)	15(4.7)	0.058 (2.542[0.940-6.871])

Hormone use	3(5.6)	11(3.4)	0.448 (1.652[0.446-6.127])
PPI use	27(50.0)	153(47.8)	0.766 (1.092[0.613-1.943])
